# A Semi-Automated Image Analysis Procedure for *In Situ* Plankton Imaging Systems

**DOI:** 10.1371/journal.pone.0127121

**Published:** 2015-05-26

**Authors:** Hongsheng Bi, Zhenhua Guo, Mark C. Benfield, Chunlei Fan, Michael Ford, Suzan Shahrestani, Jeffery M. Sieracki

**Affiliations:** 1 Chesapeake Biological Laboratory, University of Maryland Center for Environmental Science, Solomons, Maryland 20688, United States of America; 2 Shenzhen Key Laboratory of Broadband Network & Multimedia, Graduate School at Shenzhen, Tsinghua University, Shenzhen, P.R. China; 3 Department of Oceanography and Coastal Sciences, Louisiana State University, Baton Rouge, Louisiana 70803, United States of America; 4 Biology Department, Patuxent Environmental & Aquatic Research Laboratory, Morgan State University, Saint Leonard, Maryland 20685, United States of America; 5 National Oceanic and Atmospheric Administration, Silver Spring, Maryland 20910, United States of America; 6 SR2 Group LLC, Laurel, Maryland 20707, United States of America; National Taiwan University, TAIWAN

## Abstract

Plankton imaging systems are capable of providing fine-scale observations that enhance our understanding of key physical and biological processes. However, processing the large volumes of data collected by imaging systems remains a major obstacle for their employment, and existing approaches are designed either for images acquired under laboratory controlled conditions or within clear waters. In the present study, we developed a semi-automated approach to analyze plankton taxa from images acquired by the ZOOplankton VISualization (ZOOVIS) system within turbid estuarine waters, in Chesapeake Bay. When compared to images under laboratory controlled conditions or clear waters, images from highly turbid waters are often of relatively low quality and more variable, due to the large amount of objects and nonlinear illumination within each image. We first customized a segmentation procedure to locate objects within each image and extracted them for classification. A maximally stable extremal regions algorithm was applied to segment large gelatinous zooplankton and an adaptive threshold approach was developed to segment small organisms, such as copepods. Unlike the existing approaches for images acquired from laboratory, controlled conditions or clear waters, the target objects are often the majority class, and the classification can be treated as a multi-class classification problem. We customized a two-level hierarchical classification procedure using support vector machines to classify the target objects (< 5%), and remove the non-target objects (> 95%). First, histograms of oriented gradients feature descriptors were constructed for the segmented objects. In the first step all non-target and target objects were classified into different groups: arrow-like, copepod-like, and gelatinous zooplankton. Each object was passed to a group-specific classifier to remove most non-target objects. After the object was classified, an expert or non-expert then manually removed the non-target objects that could not be removed by the procedure. The procedure was tested on 89,419 images collected in Chesapeake Bay, and results were consistent with visual counts with >80% accuracy for all three groups.

## Introduction

A central goal of plankton ecology is to understand the spatial and temporal dynamics of planktonic organisms and how they overlap and interact with their environments. While nets remain the primary sampling tool for zooplankton, imaging systems provide fundamental measurements and observations that enhance our understanding of key physical and biological processes at small scales [1]. In the past three decades various optical technologies capable of imaging zooplankton have been developed, including bench-top type imaging systems such as ZooScan and FlowCAM [2–4] as well as *in situ* systems such as the Video Plankton Recorder (VPR) [5, 6], Underwater Vision Profiler (UVP) [7, 8], ZOOplankton VISualization system (ZOOVIS) [9], the Lightframe On-sight Keyspecies Investigate System (LOKI) [10], Shadow Image Particle Profiling Evaluation Recorder (SIPPER) [11, 12], and the In Situ Ichthyoplankton Imaging System (ISIIS) [13]. While the technical advances in hardware allow the deployment of imaging systems under various environmental conditions, including estuarine systems [14], extracting useful information from the large numbers of acquired images remains a major challenge [15–17].

The basic steps to extract information from images acquired by different systems are similar. The first step is to segment objects, i.e., identify pixels in an image that share certain characteristics and locate and extract objects [18]. After the object is identified, a set of feature descriptors, e.g., length, area, histogram of oriented gradients (HOG), are selected to describe the object [19]. Then the segmented objects are mapped from unlabeled instances to classes using a classifier. In the past two decades, several approaches have been developed to process zooplankton images. For example, Culverhouse et al. [20] used the Artificial Neural Network (ANN) and the Radial Basis Function classifier along with the multi-layer perceptron classifier to identify dinoflagellates from images acquired under a microscope. Sieracki et al. [4] developed a method based on fluorescence and size to separate microzooplankton. Gorsky et al. proposed a procedure using a random forest classifier to process images from ZooScan [21]. Ye et al. [22] applied a Bayesian approach to process images acquired from ZooScan. Hu and Davis [23, 24] developed a package for the VPR using the Support Vector Machine (SVM) classifier. Similar procedures have been applied with other systems, e.g., Shadow Imaging Particle Profiler and Evaluation Recorder (SIPPER) [25]. However, these methods are designed for images acquired either under controlled conditions or from *in situ* imaging systems within clear waters, where images are more consistent and of high quality.

**Fig 1:**
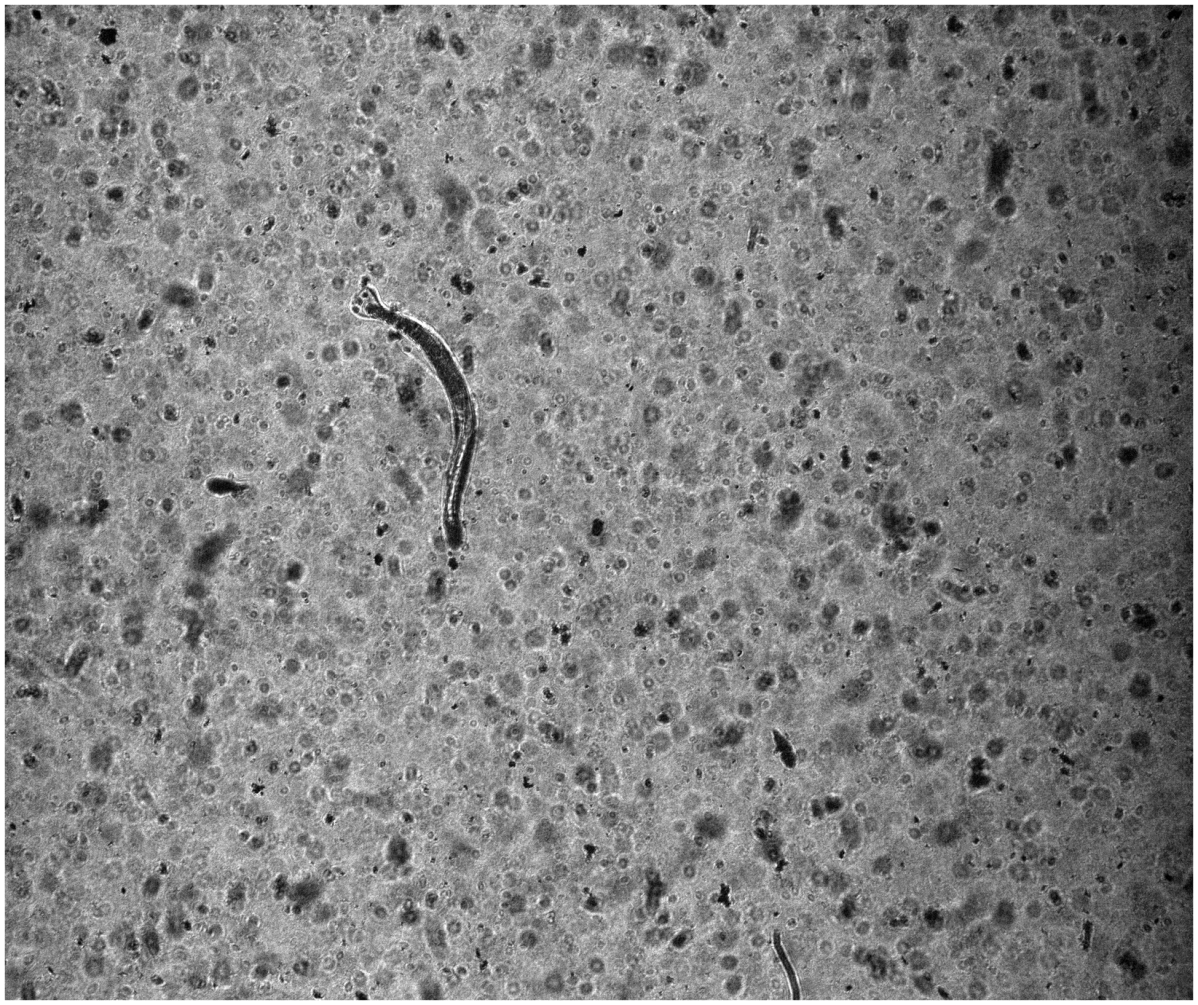
An example of a raw image collected by the ZOOplankton VISualization system in Chesapeake Bay, showing the large amount of particulates along with an arrow worm.

Processing images acquired from highly turbid waters remains a major challenge. First, reduced visibility in high-turbidity waters rapidly attenuates and scatters light, which restricts the capability of acquiring high quality images, e.g., non-uniform background illumination and low contrast (Fig 1).This makes it difficult to reliably isolate Regions of Interest (ROIs) or objects from the background. Second, the high numbers of particles in each image (Fig 1) often leads to large amounts of segmented objects, and it is difficult to classify them into proper classes. The first challenge is particularly problematic for imaging systems using scattered light sources, which attenuates rapidly in high-turbidity waters. However, it could be partially overcome using shadowgraph imaging, an approach that has been employed with ZOOVIS, ISIIS and SIPPER. Bi et al. [14] demonstrated that ZOOVIS could acquire images with acceptable quality in the middle section of Chesapeake Bay during the summer months.

In the present study, we intend to develop a procedure to process the relatively “noisy” and complex images from turbid waters, like Chesapeake Bay, because the existing procedures are designed for images collected from waters with high clarity and require a considerable amount of effort to adapt them to “noisy” images. The procedure includes four steps: segmenting ROIs, ROI denoising, feature descriptors, and lastly, taxonomic classification using the SVM.

## Methods and Materials

### Study site and sampling

The Chesapeake Bay is the largest estuary in the United States, extending approximately 320 km from its head to its mouth near Virginia Beach, Virginia. We deployed ZOOVIS in the middle portion of Chesapeake Bay near the Patuxent River mouth (longitude range: 76°18´W—76°20´W, latitude range: 38°20´N—38°23´N) on October 13, 2011. No specific permissions were required for these locations/activities. The location is not privately-owned or protected in any way. The field studies did not involve endangered or protected species. The turbidity ranged from 4–8 NTUs in the surveyed region and the Secchi depth was less than 2 m. The vessel steamed at 1 m s^-1^ and wire on the winch was paid out, or hauled back at 0.15 m s^-1^ to deploy ZOOVIS along an undulating (tow-yo) trajectory. All tows were within 2 m from the bottom to avoid possible damage to ZOOVIS and within ~ 1 m from the surface to avoid imaging within the bubble field of the ship’s wake. Grayscale images, 2448 by 2050 pixels with pixel resolution ~10 μm, were acquired at approximately 15 Hz.

In total, 89,419 images were acquired during the deployment. Images acquired within the study area were generally darker with relatively low contrast, so it was difficult to separate organisms into detailed taxonomic groups. Meanwhile, the abundances of some organisms were rare within the sampling area, e.g., larval fish and mysids, it was difficult to separate these organisms into proper taxonomic groups with enough individuals to build a proper library for classification. In the present study, all the images were visually counted and organisms were classified into categories: arrow-like organisms including chaetognatha and larval fish, copepod-like organisms including copepods, amphipods, mysids, and other crustaceans and gelatinous zooplankton including ctenophores and hydromedusae. In the present study, the semi-automatic procedure was developed in MATLAB 8.3 using the computer vision toolbox and statistical toolbox (The MathWorks Inc., Natick, MA, 2014).

### Thresholding grayscale to binary images and segmenting ROIs

**Fig 2:**
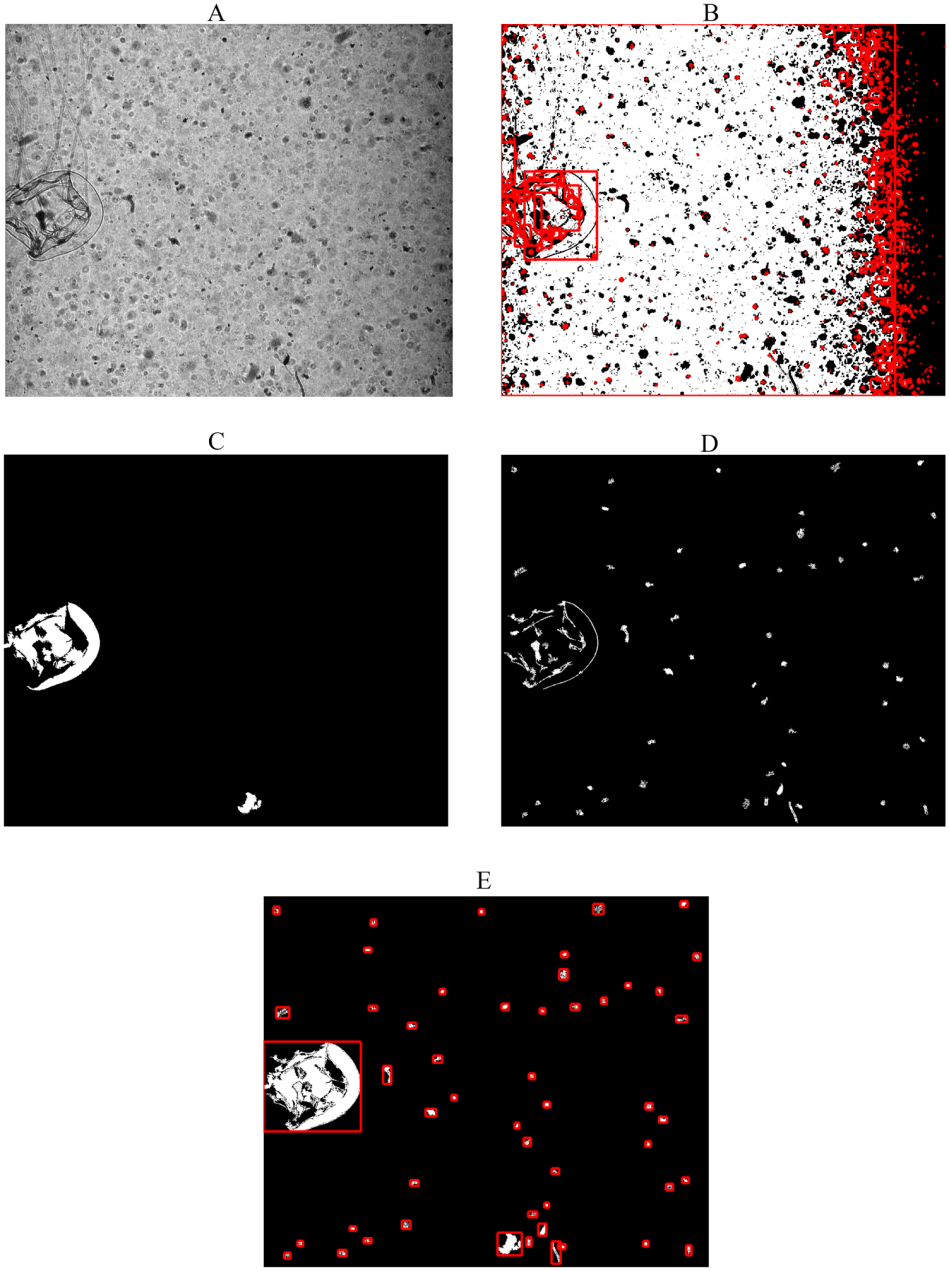
An example of a raw image collected by the ZOOplankton VISualization (ZOOVIS) system in Chesapeake Bay, and results from various thresholding approaches: A ZOOVIS image showing a hydromedusa, copepods and other particulates. **B** Binary image from the global threshold approach showing the amount of segmented objects, **C** Binary image from Maximally Stable Extremal Regions (MSER) approach showing hydromedusa and other large particulates, **D** Binary image from the customized adaptive thresholding approach showing copepods and other small particulates, and **E** Final combined binary image with red boxes showing the segmented objects.

Due to the non-uniform illumination over the field of view, it is difficult to use a single global threshold value to convert the grayscale images to binary images, i.e., all the pixels below the threshold value are black and all those above or equal are white. Global threshold approaches can cause two types of problems. First, large gelatinous zooplankton, are often segmented into separate objects, and it is time-consuming and complex to merge these parts together after segmentation. Secondly, (Fig 2A and 2B) small organisms like copepods are often missed (Fig 2B).

For large ROIs (> 5000 pixels, ~0.5 mm^2^), we applied Maximally Stable Extremal Regions (MSER) to identify connected areas characterized by similar intensity and surrounded by contrasting backgrounds [26, 27]. The basic concept of MSER is similar to thresholding, except that MSER only selects the regions which remain nearly the same over a range of thresholds. The number of areas identified largely depends upon the step size between the intensity threshold levels, and the maximum area variation between extremal regions at varying intensity thresholds. Large gelatinous zooplankton, with both transparent and more opaque body parts had different intensities, so we specified a relatively high area variation and a small step size to maintain their integrity (Fig 2C). In doing so, the procedure returned a large amount of small ROIs, so that in the present study this procedure was only applied to ROIs with > 0.5 mm^2^.

We also developed an adaptive threshold approach which worked well on small organisms. Considering the gray level distribution of images were different among individual images, we first determined the range of gray levels (Eq 1). Specifically, the variance between the foreground and background was computed for the original image based on Otsu’s method [28], and then the variance was calculated using a smoothed histogram of gray levels with a window size of 5.

$$S=\text{arg}\;\underset{T}{\text{max}}\{W_{0}\left(T\right)*\left(\mu_{0}-\mu_{t}\right)*\left(\mu_{0}-\mu_{t}\right)+W_{1}\left(T\right)*\left(\mu_{1}-\mu_{t}\right)*\left(\mu_{1}-\mu_{t}\right)\}$$ (1)

Where, *S* is the variance, *T* is a global threshold ranging from 0 to 255. *w*_0_(*T*) and *w*_1_(*T*) are the percentage of foreground and background pixels in the full image, *μ*_0_ and *μ*_1_ are the mean gray value of foreground and background pixels respectively, and *μ*_*t*_ is the mean gray value of the full image.

Then the Sauvola adaptive thresholding approaches [29] were used to convert grayscale images to binary images (Eq 2).

$$T\left(i,j\right)=m\left(i,\;j\right)\left(1+k*\left(\frac{S\left(i,\;j\right)}{R}-1\right)\right)$$ (2)

Where *m*(*i*, *j*) is the average and standard deviation value of the sliding window, *k* is a positive value based on the variance between foreground and background, and *R* is the maximum value of the standard deviation (*R* = 128 for a grayscale image) with the sliding window size as 77*77 pixels (~ 3% of image size). In present study, we tested the adaptive algorithm on a set of 35 pre-selected images which had different a number of copepods in each of them. By varying *k* and visualizing the segmented ROIs, the number of missing copepods, i.e., copepods were not segmented, were recorded and then k was determined by identifying the value with the minimum number of missing copepods. In the present study, *k* was set at 0.6.

The adaptive threshold procedure performed well for small organisms such as copepods, which often have a solid darker foreground. Yet for larger organisms, such as hydromedusa, the procedure most often divided them into pieces (Fig 2D). Consequently, this lead us to combine two binary images, to get a final binary image. From the combined binary image the connected components were then identified. The pixel indexes for each connected-component image region, along with other statistical properties such as length of major and minor axes, the area of each object and bounding box were extracted. Each connected component was then treated as a ROI, cropped from the original image, and saved for the next step.

### ROI denoising and feature descriptors

**Fig 3:**
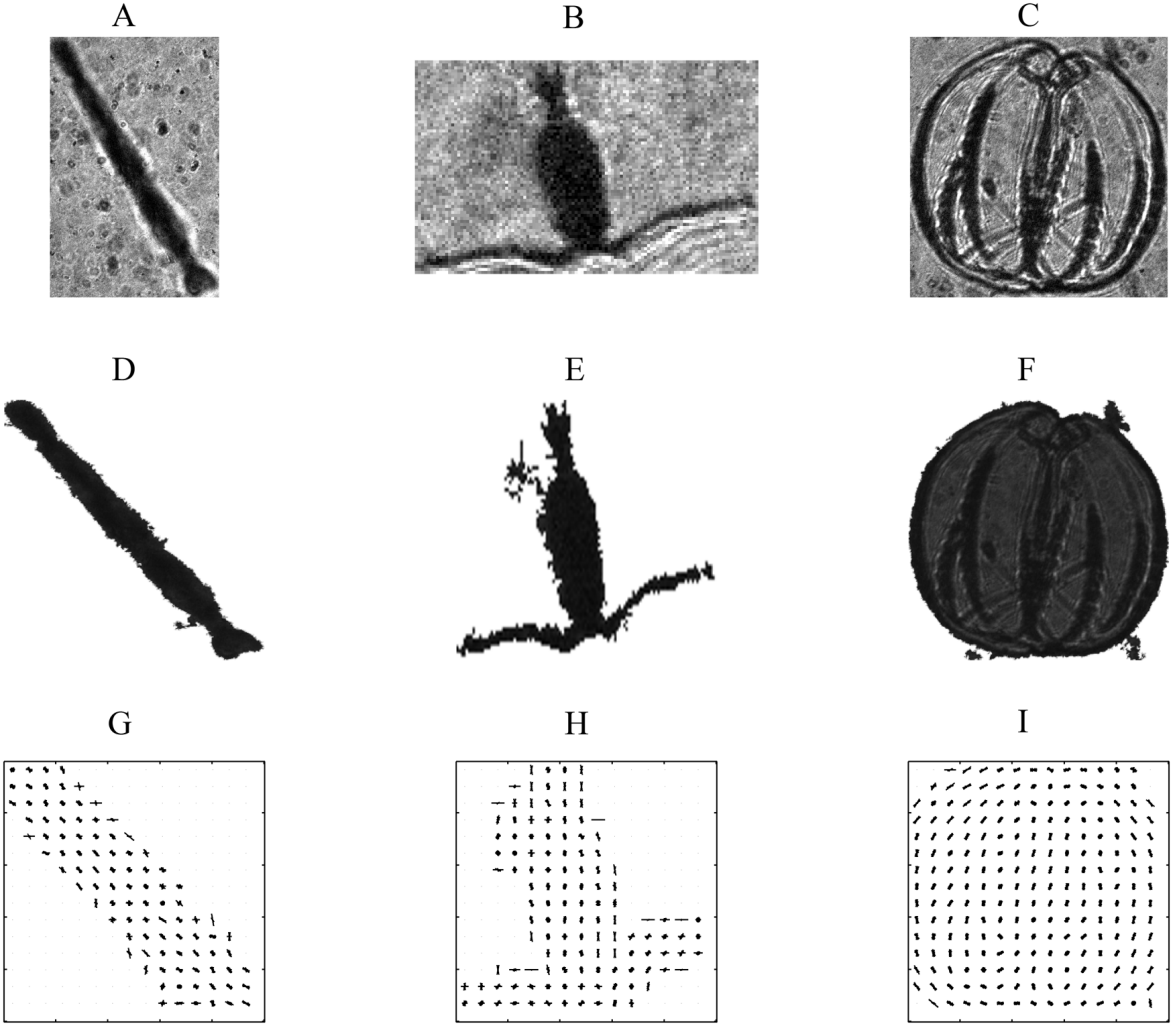
Examples of model species and construction of feature descriptor. **A**—**C**: Examples of segmented arrow-like, copepod-like, and gelatinous zooplankton, **D**—**E**: standardized (250 by 250 pixels) denoised cropped images, and **F**—**H**: Histogram of Oriented Gradients feature descriptors for three categories.

The above segmentation procedure intended to crop a ROI with a rectangular bounding box to contain a single object, however, there were often multiple objects in an individual ROI (Fig 3A–3C). To filter out “bad components” and exclude them from the list of components, the ROI was first converted to a binary image using the global threshold method. The size of ROI was relatively small relative to the entire image (2448 by 2050 pixels), consequently its background tended to be more uniform. We chose to use the global threshold approach. Once the ROI was converted to binary format, the connected components were identified. Grayscale values for the largest connected component were extracted from the original ROI and the rest of the connected components were assigned with the average gray value. We used the gray values from the original ROI instead of the binary values to retain internal structure, with special regard to gelatinous zooplankton, and then we constructed texture features for each ROI.

To analyze the patterns in the segmented ROIs and classify them into different classes, the ROIs were normalized. Then, Histogram of Oriented Gradients (HOG) features [30, 31] were constructed to describe the shape for each ROI. To construct a HOG feature descriptor, each normalized ROI was decomposed into small, squared cells so that each cell had 16*16 pixels. Then, each cell was represented by a histogram of edge orientations and in the present study we set the number of orientation histogram bins to 9 (Fig 3G–3I). The cell size was determined by varying the HOG cell size, 8*8, 16*16, and 32*32, visualizing the results, and then examining the effect of the cell size on the amount of shape information. Increasing and decreasing cell sizes were potentially useful for capturing large-scale and small-scale spatial features, respectively.

### SVM classifier

Images acquired under laboratory controlled conditions or within clear waters tend to have much less non-target objects, i.e., non-target objects are the minority and target objects are the majority class. In existing approaches, ROIs are directly classified into different classes with all non-target objects lumped together as one class. Whereas in images acquired from turbid waters, non-target objects, the majority class, often significantly outnumber the target objects, the minority class. In the present study, the target objects in each image made up < 5% of the total segmented objects. Non-target objects can take various forms and certain types of non-target objects are similar to a specific group of organisms. For example, air bubbles in near surface water generated by boat propellers, are often similar to jellyfish in terms of shape and size and some particulates are similar to arrow worms. Therefore, we designed a two-step procedure using SVM techniques.

Representative images for each taxon were selected and the ROIs were manually cropped which served as the target objects in the library. The SVM classifier (described next) was first trained using the library. Each segmented ROI was first re-sized to 250*250 pixels (2.5*2.5 mm^2^), the same order of the size of a copepod, but slightly larger. The HOG feature descriptor was constructed for each ROI and compared against the target objects. Then each segmented ROI, regardless of whether or not it contains a target object, will be classified into one of three classes: gelatinous zooplankton, arrow-like, and copepod-like. In this step, some ROIs were actually classified into more than one class.

In the second step of classification, we constructed a specific classifier for each group. Representative images for each taxon were selected and the ROIs were manually cropped, which served as the target objects in the library. A SVM classifier was trained for each class. Similar to the first step, the ROI was normalized to 150*150 pixels (1.5*1.5mm), about the size of a large copepod, *Eurytemora affinis*, a common species in the Bay. Then HOG feature descriptors were constructed and compared against the library. In this step, the SVM classifier is a group specific binary classifier (true or false) for each taxon.

The SVM is a relatively new learning method used for binary classification. In contrast with classical statistical approaches where the dimensionality of a feature’s space is decreased to control the performance, the SVM dramatically increases dimensionality, relying on large margin factors or supporting vectors [32, 33]. In short, the SVM discriminative classifier is defined by a hyperplane, instead of a single line to separate data perfectly into two classes. Furthermore, the identified hyperplane has an equal distance to the two classes. SVMs are inherently two-class classifiers. To extend this approach to multiclass classification, the most common way is to build one-versus-rest classifiers (commonly referred to as “one-versus-all”), and to choose the class which classifies the test datum with the greatest margin. We used a simple linear classifier, *f*(*X*) = *W* ^*T*^ *X*+*b* to map each ROIs to different classes, where W is a weight vector, X is a feature vector, b is the bias.

The SVM classification has been applied to plankton classification by Hu and Davis [23] and Luo et al. [34, 35]. In the present study, there are three classes that constitute this step. First, each model species or ROI was translated into one observation with paired values: a vector of x and a predicted value of y, where *x* is a vector containing the HOG descriptors, and y is the predicted value. For example, let the vector *x* contain information for copepods; if the ROI contains a copepod, *y* would be 1 and if the ROI did not contain a copepod *y* would be 0. In the first step, the training library included the following images; 80 arrow-like, 65 copepod-like, and 65 gelatinous zooplankton. These model images represent organisms with different shapes, sizes, and orientations.

**Fig 4:**
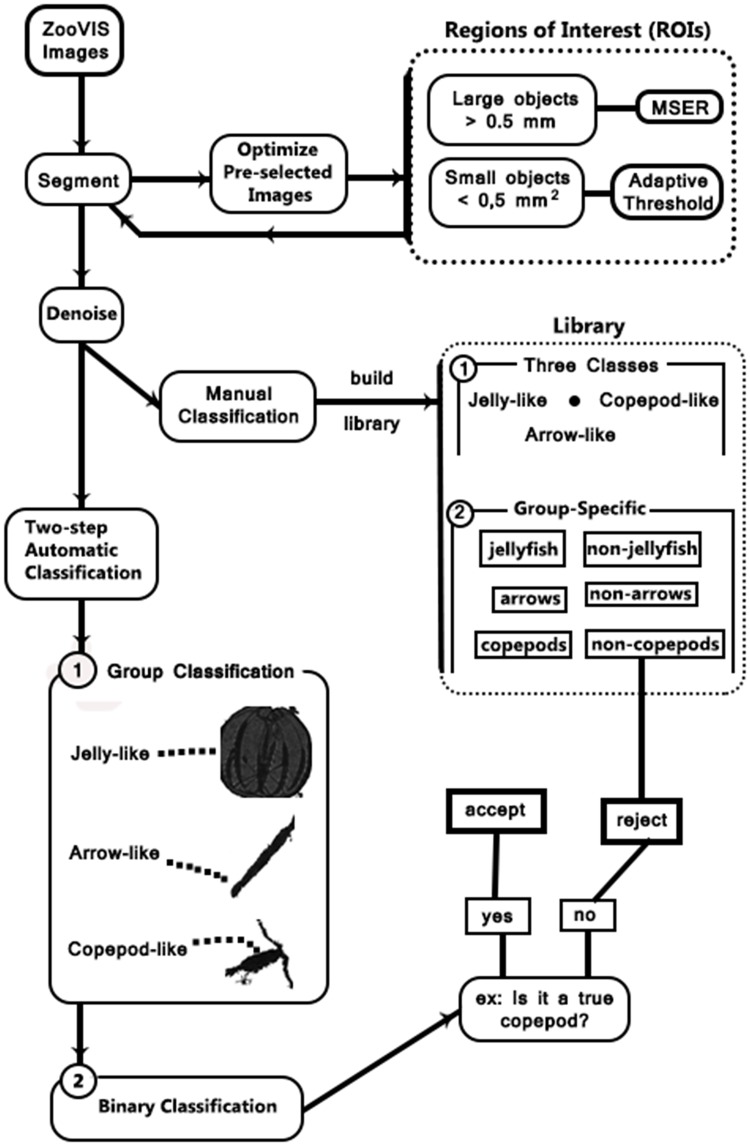
A schematic flow chart showing the proposed image processing procedure.

In the second step, each ROI was passed through a group-specific binary SVM classifier trained to separate target and non-target objects. For example, the library for copepod-like objects includes 65 ROIs that contain a copepod and 985 ROIs that do not. Similar classifiers were built for arrow-like organisms and gelatinous zooplankton. These classifiers could be built through a reiterative process. For example, our library for the copepod-like group initially only contained copepods and a few non-copepods. We applied the procedure to 400 randomly selected images and visually examined the resulted ROIs, which all were supposed to contain a copepod. Those ROIs that did not contain a copepod were moved into the library as non-target objects. The schematic flowchart in Fig 4 illustrates the proposed procedure.

### Validation

The performance of the classifier is often evaluated by a confusion matrix showing the predicted and actual classifications [19]. Through this matrix, various measurement can be calculated. In the present study, we were not able to track non-target objects classified by the classifier because in general there were hundreds, sometimes thousands, of non-target objects within each individual image (89,419 images in total). Furthermore, the computational demands to write out those non-target objects, classified by the classifier, and to then perform visual counts is too high. Therefore, we only calculated the true-positive rate, the rate to which target objects were classified as target objects by the classifier. The false positive rate, the rate by which non-target objects were classified as target objects by the classifier, and precision, the percentage of true-target objects in the target objects classified by the classifier.

We performed visual counts on over 89,419 images to get the total number of target objects, and then we examined the target objects identified by the classifier to separate true-target object and false-target objects. All images were manually visualized and organisms within each image were enumerated by one person (H. Bi) to reduce potential inconsistency.

## Results

Out of the 89,419 images, a total of 7026 copepod-like organisms, 116 gelatinous zooplankton, and 146 arrow-like organisms were visually counted. Copepod-like organisms (primarily copepods) were encountered in 5372 (~6%) images, gelatinous zooplankton (primarily *Mnemiopsis leidyi* and *Liriope tetraphylla*) in 115 (~0.13%) images and arrow-like organisms (primarily chaetognatha) in 142 (~0.16%) images. Note that many organisms such as the large gelatinous zooplankton and arrow-like organisms in particular, were distinguishable but out-of-focus, which could lead to an overestimation of their abundances because imaging volume only considered in-focus organisms.

**Fig 5:**
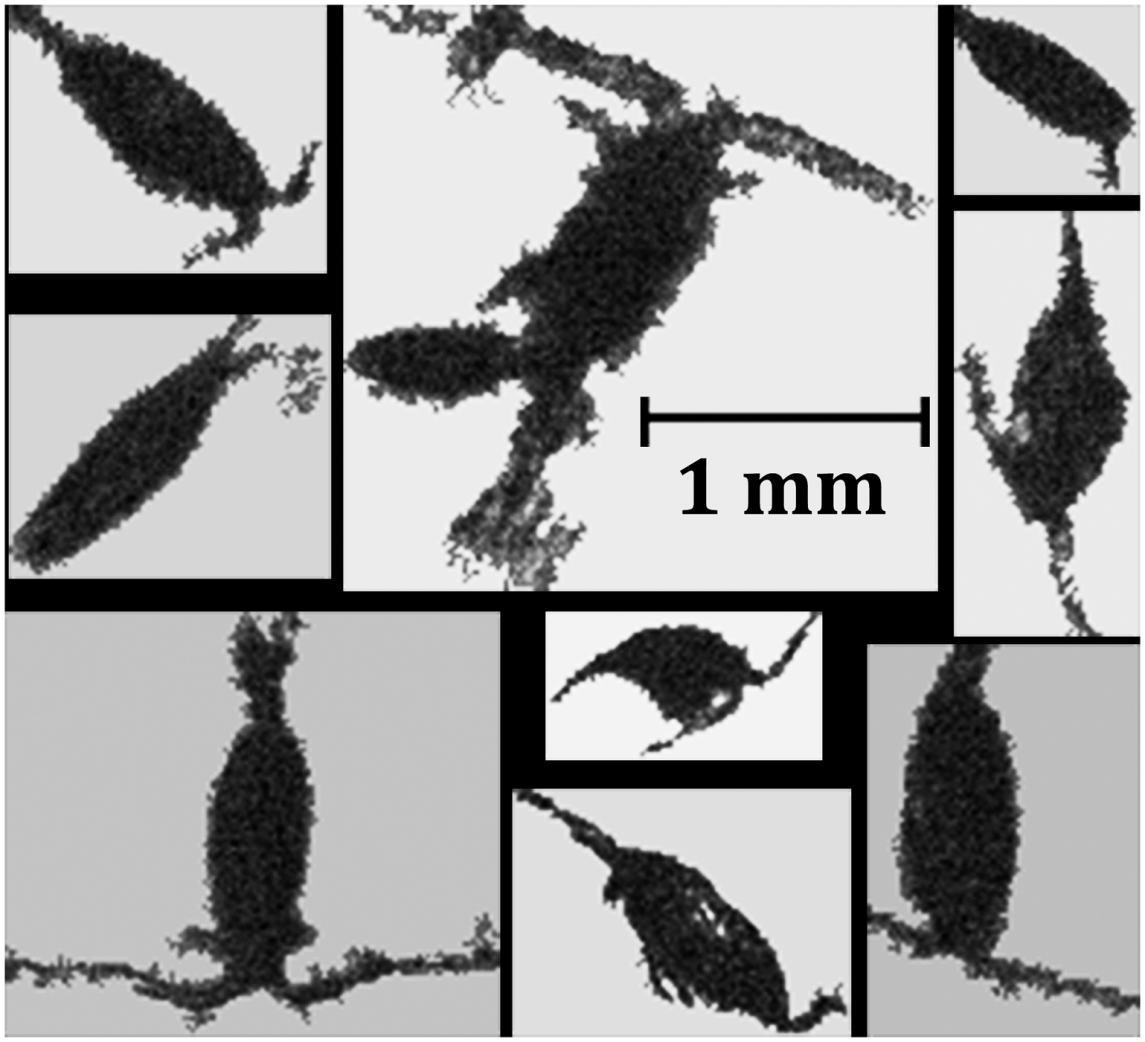
Example of copepods extracted with different sizes and orientations using the computer from the semi-automated procedure. Note the cropped images were denoised and then classified by the classifier.

**Fig 6:**
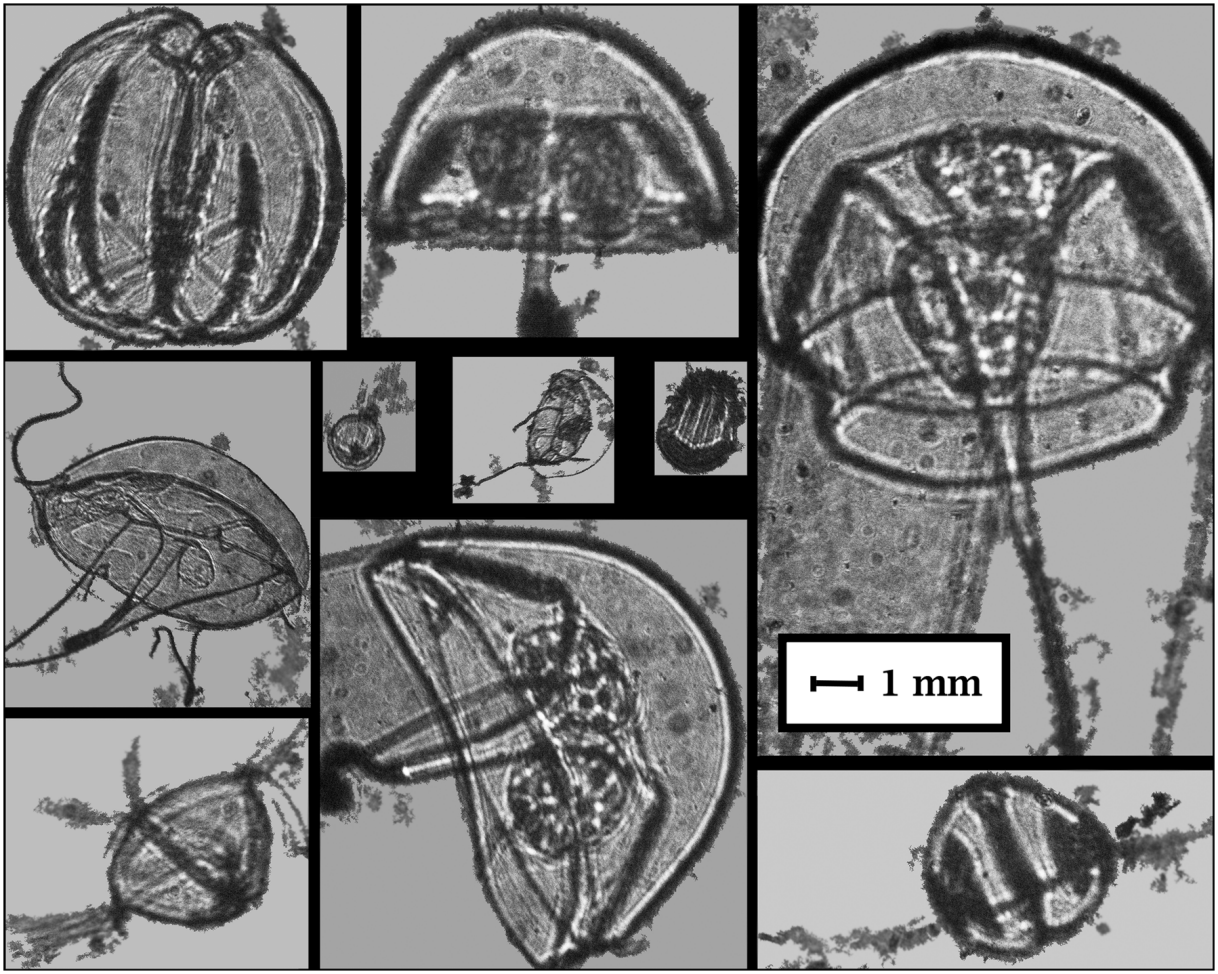
Example of ctenophores and hydromedusae with different shapes and sizes extracted using the semi-automated procedure. Note the cropped images were denoised and then classified by the classifier.

The semi-automated procedure yielded 6293 copepod-like organisms from 5333 (~6%) images, 92 gelatinous zooplankton from 92 (~0.10%) images, and 128 (~0.14%) arrow-like organisms from 119 images. The number of organisms classified by the semi-automated procedure tended to be lower than the visual counts with copepod-like organisms (~10% lower), gelatinous zooplankton counts were ~20% lower, and arrow-like organisms were ~13% lower. The overall true-positive rate was >80 for all three classes. The procedure performed well on copepod-like organisms and extracted copepods with different orientations and sizes (Fig 5). The semi-automated program was capable of extracting a wide range of gelatinous zooplankton with different sizes (Fig 6), but gelatinous zooplankton remained the main challenge for the program because some of them were segmented into different parts, although the duplicated parts were removed from the result in the present study. Overall the false positive rate appeared to be closely related to how representative the model species was. Note that the procedure was initially tested with 7 arrow-like organisms and 26 gelatinous zooplankton and resulted with ~30–40% of target objects being misclassified as non-target objects for both gelatinous zooplankton and arrow-like organisms.

## Discussion

### 1. Challenges

Like any other existing approach, the first set in the current procedure is isolating objects from the background which turns out to be very challenging. Images acquired from turbid waters are often characterized by non-uniform background and low contrast ratios between objects and background. Estuarine systems are often characterized by distinct vertical structures, often with particle-laden, low-salinity water on top of the relatively clear high-salinity coastal water. In the present study, the imaging system was towed with an undulated trajectory and resulted in images with different qualities. Furthermore, when the system was towed near surface, images often contained low amounts of air bubbles generated by boat propellers. The hybrid procedure developed here is more consistent in terms of isolating individuals. However, there are still a few issues. First, it is difficult for the procedure to separate large organisms on the edge from the background because the edge tends to be dark due to nonlinear illumination. Second, when an organism is connected with another object or organism, the procedure will segment them as one ROI. Finally, some organisms will be cut into various parts, gelatinous zooplankton in particular, due to different intensities of different body parts causing duplicated counts.

Most existing approaches in plankton imaging recognition use a flat multi-class classifier in which non-target objects are often pooled together as one class and the amount of non-target objects tend to be smaller than the present study. In turbid waters, there are often large amounts of particulates, hundreds if not thousands in each image, in the water column, especially in estuarine waters, which can exhibit diverse morphology similar to living organisms. This will cause problems in two different aspects. First, the presence of a large number of non-target objects will cause some statistical problems which could be biased towards non-target objects [36]. Second, non-target objects with diverse morphology make it difficult to separate them from the target objects. The magnitude of this problem is contingent on the amount of detritus in the water column. In the present study, we customized a two-step classifier. In the first step, all objects including both target and non-targets will be separated into three different classes based on their shapes. The rationale for this step is that some non-target objects are similar to a specific group of organisms, e.g., air bubbles versus jellyfish and long particulates versus arrow worms. Once objects are classified into different categories, they will be again classified into group-specific binary classifiers. In this second step, the majority of non-target objects are removed.

### 2. Performance of the procedure

The proposed procedure satisfies our need to process large amounts of images acquired from highly turbid waters with a precision > 80% for all three categories. There were very few non-targeted objects that occurred in the gelatinous zooplankton and arrow-like groups, however the number of non-target objects tended to be high in the copepod-like group. The relatively high false positive rate related to the copepod-like group was due to the different orientations and sizes of copepod-like organisms, which make it difficult to distinguish them from the particulates in various forms. For the gelatinous zooplankton group, there is still duplication caused by the segmentation procedure, because their body parts (hard tissue versus soft tissue) tended to have different intensities. To increase the precision, we performed a visual examination on the segmented ROIs that were classified as target objects by the classifier, to remove the false positives in each group.

The performance of the semi-automated procedure is a compromise between the true-positive rate and false positive rate. Lowering the false positive rate, often requires increasing the number of non-target objects in the library to better represent all non-target objects in the images and better train the classifier. This often leads to a low true-positive rate because an SVM classifier, trained on an imbalanced dataset, often produces models which are biased towards the majority class and have low performance on the minority class [36]. Chang et al. [37] also reported that balanced training had higher accuracy at recognizing rare taxa but low accuracy at abundant taxa. In the present study, the ratio of target objects (minority class) and non-target objects (majority class) in the library was ~ 8% and was consistent with the ratio in the images 1–5%. In the customized procedure, we controlled the false positive rate (<10%) and accuracy (>80%) and then manually removed the misclassified non-target objects (false positives) to increase accuracy to a maximum level.

The performance of the customized procedure for rare groups is closely related to number of model species in the library during the binary classification step. The number of model species, i.e., how representative the model species are, is essential to the performance of the procedure. In the present study, we found that by increasing the number of model species for rare organisms, e.g., gelatinous zooplankton and arrow-like organisms, we could significantly reduce the number of target objects which were classified as non-target objects by the classifier.

Another important factor that can affect the true-positive rate and false positive rate is the feature descriptor. The HOG descriptors were adopted to train the SVM classifier. We tested the potential of using statistical properties including size, axis length etc., and the procedure yielded a large amount of false positives because there were large amounts of particulates in the estuarine water with similar statistical features as organisms. The cell size for the HOG descriptors could have a large impact on the missing rate and error rate. Large cell size, i.e., intensity gradient over a large area, means fewer feature descriptors and coarse resolution, which leads to higher false positive and true-positive rates. To contrast this, small cell sizes mean more feature descriptors and fine resolution, which leads to lower false positive and true-positive rates.

Another important factor to consider is the processing speed, especially with the large amount of images collected in each cruise. The processing speed varies a lot depending on the contents, i.e., the number of segmented objects, in each image. However, a general rule for the SVM classification is that an increase in dimensions, including number of groups, number of model species in the library, and number of feature descriptors will slow down the processing speed. Our customized procedure was tested on a small workstation with an AMD Optimum II processor and resulted in an acceptable processing speed of 8,000–12,000 images per day, depending on the contents. Increasing the dimension could lead to better control of the error rate and missing rate at the expense of processing speed. The semi-automated procedure is a compromise between error rate, missing rate, and processing speed. However, there is no quantitative measurement to evaluate the performance due to the complex nature of image processing and classification.

**Fig 7:**
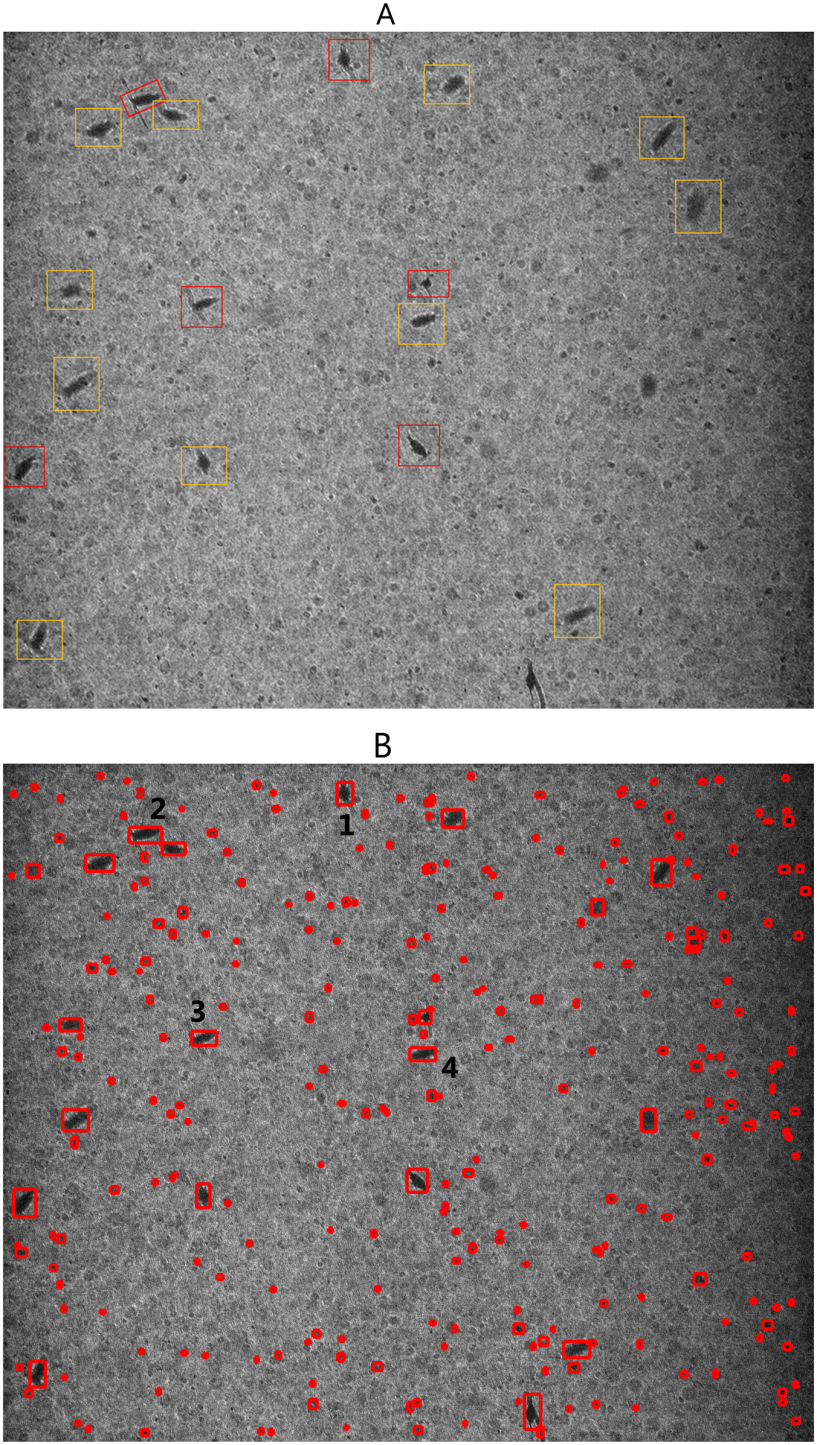
An example of image comparing visual counts and results from computer-based procedure. **A)** A raw image contains copepods showing copepods in focus indicated by red boxes and out of focus indicated by yellow boxes. **B)** The same image processed by the computer-based procedure with red boxes indicating segmented objects and resulting copepods were numbered.

The difference between visual counts and the computer-based procedure arises from two different aspects: inaccuracy in visual counts, and potential errors from the semi-automated procedure. One of the main obstacles for visual counting was to consistently separate in-focus individuals from those that were slightly out-of-focus (Fig 7a), whereas the computer-based algorithm only identifies objects with full features, e.g., copepods (Fig 7b), and is more consistent. Culverhouse et al. [38] showed that human experts were not 100% accurate in plankton identification and even trained personnel can be expected to achieve 67 to 83% self-consistency. Therefore, the difference between visual counts and the semi-automated procedure could be partially attributed to the inaccuracy in the visual counts. To better understand the performance of the computer procedure, we need to estimate the error rate in visual counts.

### 3. Conclusion and future developments

Here we have shown a robust procedure to separate objects from non-uniform background. It could be enhanced in several different ways. We can apply noise reduction techniques to enhance image quality [39, 40] or use an advanced background removal procedure to address the non-uniform illumination [41] to better separate objects from their background. While these techniques will certainly improve the performance of the procedure, the improvements come at a price of high computational demand. Given the large volume of images from the *in situ* imaging systems, it is necessary to control computational demand.

Classification is an integral part of plankton image analysis. Babbar et al. [42] showed that when the classification problem is highly unbalanced, hierarchical classifiers would perform better than flat multi-class models. In the present study, we employed a two-level hierarchical model and results are promising. In the future, we will move towards a multi-level hierarchical model and conduct comparisons among different classification models. Meanwhile, it is important to determine the proper number of classes/groups to optimize a classifier. As the number of classes increases, so do the computation demands and the false positive rate. Selecting an appropriate number of categories is therefore a balance between adequate taxonomic discrimination and classifier accuracy. Rare species will continue to be a major challenge because a well-trained classifier requires a library that can represent all objects that it will encounter. Relatively large gelatinous zooplankton are problematic, mainly due to variations in tissue opacity, which are represented with different intensities within the grayscale images, and quite often segmented into separate objects rather than as a whole organism. The development of an algorithm that equalizes the light illumination to segment gelatinous zooplankton without cutting them into multiple parts is also important.

There is also a need to determine the optimum number of classes, which is a balance between increasing precision and processing time. We need to develop a quantitative approach that produces a detailed comparison on the performance with a different number of classes. We also need to evaluate how the imbalanced classifier affects the performance of the procedure when the non-target objects makes up >95% of the segmented objects. Furthermore, we need to build training sets that are representative of the spatial and temporal diversity of zooplankton taxa present in the study area, so that appropriate training sets are available to train the classifier based on the presence of rare or particular taxa of interest.

Our work underscores the challenges involved with developing semi-automated procedures for plankton identification, especially with images collected from highly turbid estuarine waters. Regardless, it has proved to be a promising technique that was able to produce estimates similar to visual counts. We have tested different types of feature descriptors, but with limited success in terms of controlling the precision, false positive rate and processing time. Development of useful feature descriptors, e.g., fast identification of the presence of key features such as antenna for copepods, would improve the accuracy of the semi-automated procedure.

Fast and accurate image processing and classification remains a major bottleneck for the deployment of in situ plankton/ichthyoplankton imaging systems. The proposed procedure in the present study was built in Matlab. Like other existing techniques, the procedure can process images in common forms, thus it can be easily adapted to different imaging systems. As image processing theories and techniques advance, we expect new tools will continue to emerge and facilitate the development of semi-automated plankton imaging analysis and identification.

The source code is available at https://github.com/bihshlsu/ZOOVIS_image_processing.git. Raw images are available upon request. Please contact Hongsheng Bi at hbi@cbl.umces.edu.
